# Supplementary material for: Comparative genomics provides new insights into the diversity, physiology, and sexuality of the only industrially exploited tremellomycete: Phaffia rhodozyma
Source: BMC Genomics. 2016 Nov 9;17:901. doi: 10.1186/s12864-016-3244-7 (PMC5103461; doi:10.1186/s12864-016-3244-7)
Supplement: Additional file 6: — List of orphan genes with links to PFAM (related to Additional file 1: Table S1). (ZIP 1428 kb) [file 12864_2016_3244_MOESM6_ESM.zip › BLAST_HTML_FTR/G02098_P.html]

BLAST Search Results


```
BLASTP 2.2.27+


Reference:
Stephen F. Altschul, Thomas L. Madden, Alejandro A. Schäffer,
Jinghui Zhang, Zheng Zhang, Webb Miller, and David J. Lipman (1997),
"Gapped BLAST and PSI-BLAST: a new generation of protein database
search programs", Nucleic Acids Res. 25:3389-3402.


Reference for
composition-based statistics:
Alejandro A. Schäffer, L. Aravind, Thomas L. Madden, Sergei
Shavirin, John L. Spouge, Yuri I. Wolf, Eugene V. Koonin, and
Stephen F. Altschul (2001), "Improving the accuracy of PSI-BLAST
protein database searches with composition-based statistics and
other refinements", Nucleic Acids Res. 29:2994-3005.


Database: nr
           71,551,133 sequences; 26,053,659,533 total letters


Query= G02098_P

Length=728
                                                                      Score     E
Sequences producing significant alignments:                          (Bits)  Value

emb|CED82180.1|  hypothetical protein [Xanthophyllomyces dendrorh...  1446    0.0  
ref|XP_012764360.1|  osmiophilic body protein [Plasmodium reichen...  45.4    0.26 


 >emb|CED82180.1| hypothetical protein [Xanthophyllomyces dendrorhous]
Length=727

 Score = 1446 bits (3743),  Expect = 0.0, Method: Compositional matrix adjust.
 Identities = 727/727 (100%), Positives = 727/727 (100%), Gaps = 0/727 (0%)

Query  1    MNDPLQDYPSLGDPSLGVDLAQDPHPSFDSSGLHHDHHHHHHHHHHLHNDHVNHHESVQE  60
            MNDPLQDYPSLGDPSLGVDLAQDPHPSFDSSGLHHDHHHHHHHHHHLHNDHVNHHESVQE
Sbjct  1    MNDPLQDYPSLGDPSLGVDLAQDPHPSFDSSGLHHDHHHHHHHHHHLHNDHVNHHESVQE  60

Query  61   LPSHHDHLNHDHLDHHSHSHEPGQPHDENDHDNRDENHQSIDLPMEEMQRALEQHHPSFE  120
            LPSHHDHLNHDHLDHHSHSHEPGQPHDENDHDNRDENHQSIDLPMEEMQRALEQHHPSFE
Sbjct  61   LPSHHDHLNHDHLDHHSHSHEPGQPHDENDHDNRDENHQSIDLPMEEMQRALEQHHPSFE  120

Query  121  RQLQQLQDAHNRQQQQQQQQQDQENGVVRADEDNQQKQQRQRQQQHLAYHHEDGSISLPE  180
            RQLQQLQDAHNRQQQQQQQQQDQENGVVRADEDNQQKQQRQRQQQHLAYHHEDGSISLPE
Sbjct  121  RQLQQLQDAHNRQQQQQQQQQDQENGVVRADEDNQQKQQRQRQQQHLAYHHEDGSISLPE  180

Query  181  GTFIHLEDDSGSGSHVDLEGHHSLDLNGQADQVDSTTIDGELDLEEGGGRGGGGLGGGLG  240
            GTFIHLEDDSGSGSHVDLEGHHSLDLNGQADQVDSTTIDGELDLEEGGGRGGGGLGGGLG
Sbjct  181  GTFIHLEDDSGSGSHVDLEGHHSLDLNGQADQVDSTTIDGELDLEEGGGRGGGGLGGGLG  240

Query  241  GAGAGGKKKRPKYGINQDEDRLAKIRAQGRERQRRKRERDRAQKSQDPSLILQDASQPQA  300
            GAGAGGKKKRPKYGINQDEDRLAKIRAQGRERQRRKRERDRAQKSQDPSLILQDASQPQA
Sbjct  241  GAGAGGKKKRPKYGINQDEDRLAKIRAQGRERQRRKRERDRAQKSQDPSLILQDASQPQA  300

Query  301  STSTAHGTLANRRMGLTRPGSHSGVGVVGDNSAQASANAARKKRKPNQTGQSDLSQHYSA  360
            STSTAHGTLANRRMGLTRPGSHSGVGVVGDNSAQASANAARKKRKPNQTGQSDLSQHYSA
Sbjct  301  STSTAHGTLANRRMGLTRPGSHSGVGVVGDNSAQASANAARKKRKPNQTGQSDLSQHYSA  360

Query  361  HGSPSPGPSAGGYQSSSRSARSSSTTQHILDLSAALSLHSNSTNGTGTNISSGGGDHHHH  420
            HGSPSPGPSAGGYQSSSRSARSSSTTQHILDLSAALSLHSNSTNGTGTNISSGGGDHHHH
Sbjct  361  HGSPSPGPSAGGYQSSSRSARSSSTTQHILDLSAALSLHSNSTNGTGTNISSGGGDHHHH  420

Query  421  QNHIQQHHHHHHPSESPISTGPTPNGHLFASTLLLALQMNDGLKDHLQRTLGVMETDYEN  480
            QNHIQQHHHHHHPSESPISTGPTPNGHLFASTLLLALQMNDGLKDHLQRTLGVMETDYEN
Sbjct  421  QNHIQQHHHHHHPSESPISTGPTPNGHLFASTLLLALQMNDGLKDHLQRTLGVMETDYEN  480

Query  481  MRAGIGEVFDRYVDDHNARAAAAAVAVALAEATGGEGSDADRVQDEGGEPEAEGILRMDI  540
            MRAGIGEVFDRYVDDHNARAAAAAVAVALAEATGGEGSDADRVQDEGGEPEAEGILRMDI
Sbjct  481  MRAGIGEVFDRYVDDHNARAAAAAVAVALAEATGGEGSDADRVQDEGGEPEAEGILRMDI  540

Query  541  GALSSREGSELGDRRESGKDSLEPTDLQITTTNSFDHVPNPSSIDPPSIPPSPPPLPLTR  600
            GALSSREGSELGDRRESGKDSLEPTDLQITTTNSFDHVPNPSSIDPPSIPPSPPPLPLTR
Sbjct  541  GALSSREGSELGDRRESGKDSLEPTDLQITTTNSFDHVPNPSSIDPPSIPPSPPPLPLTR  600

Query  601  EARTPSDSPPDHHSTSSLPSPNRVSFDHSKPSVRSLSPLFSVDPERDLGRGTDLDRDPDA  660
            EARTPSDSPPDHHSTSSLPSPNRVSFDHSKPSVRSLSPLFSVDPERDLGRGTDLDRDPDA
Sbjct  601  EARTPSDSPPDHHSTSSLPSPNRVSFDHSKPSVRSLSPLFSVDPERDLGRGTDLDRDPDA  660

Query  661  SNAHPVQFTLSLDDGPHHFSFGPDDLEDHSSVVATLQHLQQLQFQQLQQEAGVSPVGEAV  720
            SNAHPVQFTLSLDDGPHHFSFGPDDLEDHSSVVATLQHLQQLQFQQLQQEAGVSPVGEAV
Sbjct  661  SNAHPVQFTLSLDDGPHHFSFGPDDLEDHSSVVATLQHLQQLQFQQLQQEAGVSPVGEAV  720

Query  721  VGGNESW  727
            VGGNESW
Sbjct  721  VGGNESW  727


>ref|XP_012764360.1| osmiophilic body protein [Plasmodium reichenowi]
 emb|CDO65773.1| osmiophilic body protein [Plasmodium reichenowi]
Length=3137

 Score = 45.4 bits (106),  Expect = 0.26, Method: Composition-based stats.
 Identities = 31/134 (23%), Positives = 67/134 (50%), Gaps = 7/134 (5%)

Query  34   HHDHHHHHHHHHHLHNDHVNHHESVQELPSHHDHLNHDHLDHH--SHSHEPGQPHDENDH  91
            H+ H   H+H+   H+ +++ H+   +    HDH N D  DHH   H H   Q +   D 
Sbjct  821  HNGHMDQHNHNIDQHDHNIDQHDHNID---QHDH-NIDQHDHHMNQHDHHIDQHNHHIDQ  876

Query  92   DNRDENHQSIDLPMEEMQRALEQHHPSFERQLQQLQDAHNRQQQQQQQQQDQENGVVRAD  151
             N   +H+S +  ++  Q  + + H SF    +  ++  NR+ +++ +   +E    R +
Sbjct  877  HNHHMDHKSNNQFLQIHQNYV-RGHSSFVTISEGEENHDNRELRKKIEANLKEEWKKRFN  935

Query  152  EDNQQKQQRQRQQQ  165
            E  +Q++++++ ++
Sbjct  936  EQQEQRERKKKVEE  949


 Score = 44.7 bits (104),  Expect = 0.51, Method: Composition-based stats.
 Identities = 30/126 (24%), Positives = 50/126 (40%), Gaps = 1/126 (1%)

Query  34   HHDHHHHHHHHH-HLHNDHVNHHESVQELPSHHDHLNHDHLDHHSHSHEPGQPHDENDHD  92
             HDHH + H HH   HN H++ H    +  S++  L         HS        E +HD
Sbjct  855  QHDHHMNQHDHHIDQHNHHIDQHNHHMDHKSNNQFLQIHQNYVRGHSSFVTISEGEENHD  914

Query  93   NRDENHQSIDLPMEEMQRALEQHHPSFERQLQQLQDAHNRQQQQQQQQQDQENGVVRADE  152
            NR+   +      EE ++   +     ER+ +  +D  N   Q+   +  +       DE
Sbjct  915  NRELRKKIEANLKEEWKKRFNEQQEQRERKKKVEEDEMNETIQKHDMETSKLEKKEEVDE  974

Query  153  DNQQKQ  158
              Q ++
Sbjct  975  VTQDEE  980


 Score = 43.9 bits (102),  Expect = 0.82, Method: Composition-based stats.
 Identities = 38/163 (23%), Positives = 71/163 (44%), Gaps = 23/163 (14%)

Query  34   HHDHHHH----HHHHHHLHNDHVNHHESVQELPSHHDHLNHDHLDHHSH---SHEPGQPH  86
            H D H+H    H H+   H+ +++ H+   +   HH + +  H+D H+H    H     H
Sbjct  824  HMDQHNHNIDQHDHNIDQHDHNIDQHDHNIDQHDHHMNQHDHHIDQHNHHIDQHNHHMDH  883

Query  87   DEND-----HDNRDENHQSIDLPMEEMQRALEQHHPSFERQLQQLQDAHNRQQQQ----Q  137
              N+     H N    H S     E      E++H +  R+L++  +A+ +++ +    +
Sbjct  884  KSNNQFLQIHQNYVRGHSSFVTISEG-----EENHDN--RELRKKIEANLKEEWKKRFNE  936

Query  138  QQQQDQENGVVRADEDNQQKQQRQRQQQHLAYHHEDGSISLPE  180
            QQ+Q +    V  DE N+  Q+   +   L    E   ++  E
Sbjct  937  QQEQRERKKKVEEDEMNETIQKHDMETSKLEKKEEVDEVTQDE  979


Lambda      K        H        a         alpha
   0.311    0.129    0.382    0.792     4.96 

Gapped
Lambda      K        H        a         alpha    sigma
   0.267   0.0410    0.140     1.90     42.6     43.6 

Effective search space used: 8406690895830


  Database: nr
    Posted date:  Sep 23, 2015 12:05 AM
  Number of letters in database: 26,053,659,533
  Number of sequences in database:  71,551,133


Matrix: BLOSUM62
Gap Penalties: Existence: 11, Extension: 1
Neighboring words threshold: 11
Window for multiple hits: 40
```
